# Effects of Late Evening Snack on Cirrhotic Patients: A Systematic Review and Meta-Analysis

**DOI:** 10.1155/2018/9189062

**Published:** 2018-04-01

**Authors:** Ying-jie Guo, Zi-bin Tian, Na Jiang, Xue-li Ding, Tao Mao, Xue Jing

**Affiliations:** Department of Gastroenterology, The Affiliated Hospital of Qingdao University, Qingdao, Shandong 266003, China

## Abstract

**Background:**

Energetic effects of late evening snack (LES) on cirrhotic patients were reported recently, but there was no quantitative analysis. In this meta-analysis, we reviewed and quantified the effects of LES on energy metabolism and substrate oxidation in the patients with cirrhosis, which will be of benefit for liver cirrhosis nutritional therapy.

**Methods:**

A systematic search was conducted in PubMed, Embase, Web of Science, Elsevier, China National Knowledge Infrastructure, and Wanfang Database for relevant trials published until July 2017. These studies statistically were combined and analyzed by RevMan 5.3.

**Results:**

Fourteen trials comprising 478 cases were eligible for analysis. The results showed that the respiratory quotient value (MD = 11.09) and carbohydrate oxidation value (MD = 0.05) significantly elevated with one week or with up to three weeks of LES treatment in cirrhotic patients (*P* < 0.05). Meanwhile, the levels of serum albumin (MD = 2.98) and cholinesterase (SMD = 1.09) were increased with LES administration for three weeks or that lasting twelve weeks (*P* < 0.05). However, there was no significant improvement for the levels of alanine aminotransferase (ALT) (*P* = 0.53), aspartate aminotransferase (AST) (*P* = 0.96), and total bilirubin (TB) (*P* = 0.32).

**Conclusions:**

LES could improve the energy malnutrition state of cirrhotic patients. However, it may have little effect on reducing liver parenchymal injury indexes such as serum aminotransferase.

## 1. Introduction

Liver cirrhosis has been a serious health problem with high morbidity and mortality in the world [1]. Cirrhotic patients exhibit abnormal metabolism, including increased fat oxidation, decreased glucose oxidation, and protein-energy malnutrition (PEM), which were the main reasons leading to poor prognosis [2].

Cirrhotic patients having last eaten at 7 pm the day before will be experiencing starvation at the same level as a healthy person who has fasted for 3 days in the morning and will be experiencing reduction of nonprotein respiratory quotient (npRQ) because of an increased fat-burning rate [3, 4]. A late evening snack (LES) was recommended for patients with liver cirrhosis to improve the morning starving state [5].

Recent progressive studies showed that LES had various physiological effects, such as antihypertension, antiobesity, and antiamnesia properties and that it is helpful in maintaining a greater health-related quality of life (QOL) for patients with cirrhosis [6]. Nevertheless, the quantitative analysis of LES in cirrhotic patients is not clear, except a systematic review of trials about LES in cirrhosis patients published up to December 2011 [7]. It reported that LES was considered beneficial to decrease lipid oxidation and improve nitrogen balance. However, the results were summarized only by table form, lacking a systematic data analysis.

Recently, several new studies of potentially higher quality have been published. Being able to establish an average difference of pre-LES and post-LES in serum biochemical parameters and fuel metabolism indexes would be helpful for cirrhotic patients and clinical therapy. The quantitative analysis may provide more sufficient and more powerful evidence in the context of the current medical literature. Therefore, the objective of our study was to statistically combine these studies to make a quantitative analysis and evaluate the efficacy of LES treatment in patients with liver cirrhosis.

## 2. Methods

### 2.1. Searching Strategies

Systematic search was performed on PubMed, Embase, Web of Science, Elsevier, China National Knowledge Infrastructure, and Wanfang Database for articles published up to July 2017. The following keywords were used during the search: “late evening snack” or “nocturnal nutritional supplementation” or “nocturnal snack” or “evening snack” or “nocturnal meal” or “bedtime snack” and “cirrhosis” or “cirrhotic.” Two investigators screened titles and abstracts of all relevant articles by predetermined criteria. The full texts of potential eligible studies were cross-checked. Reference lists of all articles were scrutinized to retrieve additional literatures on this topic. There were no restrictions on publication language.

### 2.2. Inclusion and Exclusion Criteria

Articles which have the following criteria were included: (i) study design: comparisons of LES versus non-LES or pre-LES versus post-LES; (ii) study population: patients with cirrhosis, evaluated with regard to severity of cirrhosis according to Child–Pugh classification; and (iii) illustrated at least one of the outcome measures: serum albumin level (ALB), prealbumin (PAB), cholinesterase (CHE), hemoglobin (HB), alanine aminotransferase (ALT), aspartate aminotransferase (AST), total bilirubin (TB), respiratory quotient (RQ), carbohydrate oxidation rate (CHO%), protein oxidation rate (PRO%), and fat oxidation rate (FAT%). Studies excluded from the analysis were (i) trials that did not provide original data or the outcomes of interest were not reported and (ii) letters, leading articles, animal experiments, expert opinion, book sections, and case reports.

### 2.3. Data Extraction and Quality Assessment

Data extraction was performed independently by two investigators. The following information was extracted from each trial: title, the first author, study design, patient characteristics, treatment regimens, intervention details (including the composition or type of formulation used), biochemical parameters, and energy metabolism outcomes. All data was checked by a third investigator, and disagreements were resolved by discussion among all researchers. The Cochrane Collaboration's tool for assessing risk of bias [8] was used to assess the methodological quality of the randomized controlled trials (RCTs), and the Quality Assessment Tool for Quantitative Studies [9] assessed the quality of controlled pre–post studies and nonrandomized experimental studies (non-RCTs). The following domains were evaluated: selection bias, performance bias, detection bias, reporting bias, study design, confounders, blinding, data collection method, and dropouts. In both quality assessment tools, each domain will be considered as strong, moderate, or weak and studies will be classified as high, moderate, and low quality.

### 2.4. Statistical Analysis

Data was analyzed using Review Manager Software 5.3 (RevMan5.3). Statistical heterogeneity between studies was assessed by the *I*^2^ test, with *I*^2^ > 50% indicating significant heterogeneity. A random effect model was used but in the event of significant heterogeneity, a fixed effect model was used otherwise [10]. The mean differences (MD) or standardized mean differences (SMD) were presented with 95% confidence interval (CI) for the continuous data variables, which SMD was used to account for difference in measurement methods or units among included studies. Subgroup analysis was performed to identify the effects of different LES formulations.

## 3. Results

### 3.1. Search Results and Study Characteristics

**Figure 1:**
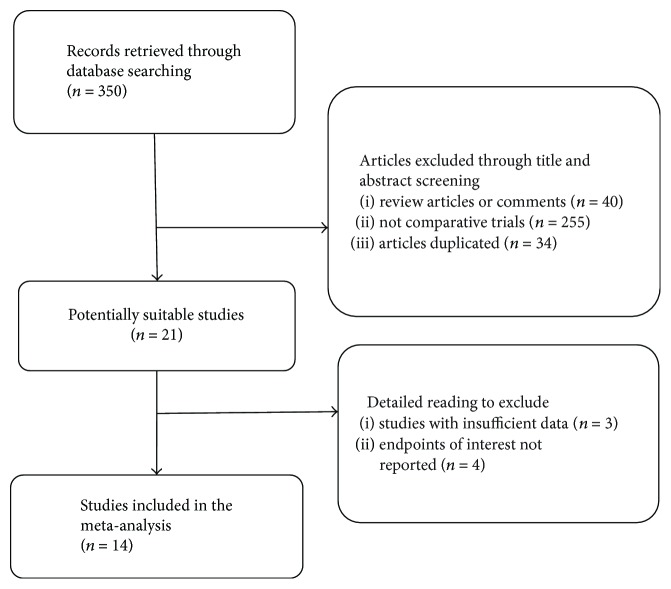
Flow diagram of study selection.

We initially identified 350 publications using the abovementioned search strategy, among which 336 articles did not meet the inclusion criteria and were subsequently excluded.  Figure 1 details the selection process. A total of 14 studies [11–24] were included in this review.

The 14 studies were published between 1997 and 2017; of these, seven trials were conducted in China [11, 13–15, 17, 19, 20], six in Japan [12, 16, 18, 21–23], and the last one in Egypt [24]. Five RCTs [11–15] and three case-control studies [16–18] compared LES to non-LES, two trials [19, 24] reported the efficacy of different doses or composition of LES in cirrhosis, and four had a pre-/postdesign and did not include a comparison group [20–23]. All studies have researched the change of biochemical and energy parameters before and after LES intervention, and pre–post intervention mean differences of these parameters will be calculated as the primary outcome. The statistical difference of LES versus non-LES was analyzed, when appropriate. Subgroup analysis will be performed based on the characteristics of LES intervention in the studies. The risk of bias assessments showed that most of these studies were of moderate quality. A description of study characteristics is given in Table 1.

### 3.2. Serum Biochemical Parameters

**Figure 2:**
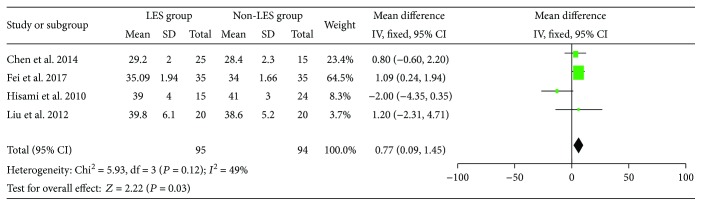
Forest plot for ALB (RCTs).

In this meta, four RCTs and eleven pre-LES versus post-LES studies evaluated the change of ALB after the administration of LES. Types and formulas of the LES were ignored when determining the total effects of LES on ALB. There was no evidence of heterogeneity between the four RCTs (*I*^2^ = 49%) and the fixed model was applied. There was significant pooled MD favoring LES versus non-LES on ALB (MD = 0.77, 95% CI: 0.09–1.45, *P* = 0.03) (Figure 2).

**Figure 3:**
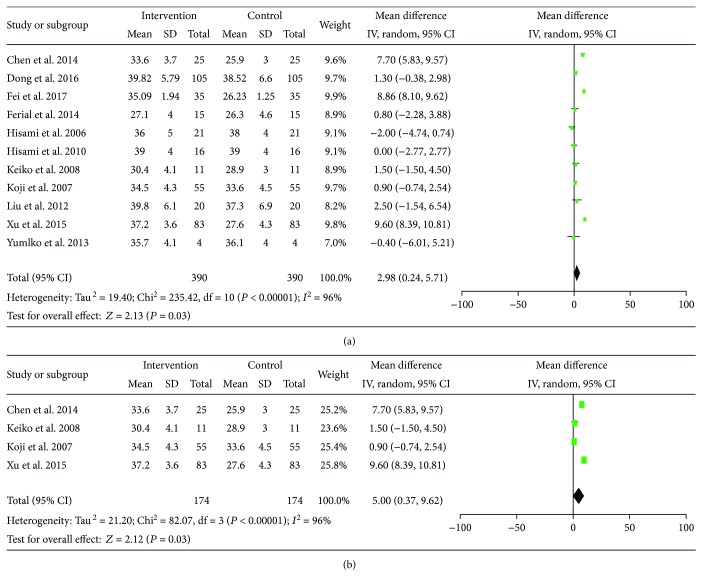
Forest plot for ALB. (a) pre–post studies; (b) subgroup analysis.

The pre-LES versus post-LES included 390 participants from 11 studies. A random effect model was used because the statistical heterogeneity was significant (*I*^2^ = 96%). Pooled results suggest that ALB increased from baseline after three–twelve weeks of LES intervention (MD = 2.98, 95% CI: 0.24–5.71, *P* = 0.03) (Figure 3).

Subgroup analysis based on the types and formulas of the LES was conducted. We analyzed the effect of high protein or branched-chain amino acid (BCAA) on ALB. Four of the fourteen studies were administered with high protein or BCAA [13–15, 21, 22, 24]; others involved mixture of various nutrients. Evidence indicates that LES, which is rich in quality protein and amino acids, has a positive effective on ALB (MD = 5.0, 95% CI: 0.37–9.62, *P* = 0.03) (Figure 3(a)). The random effect model was used with significant heterogeneity (*I*^2^ = 96%).

**Figure 4:**
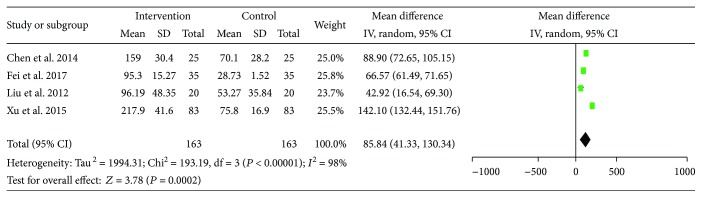
Forest plot for PAB.

PAB is an indicator estimating liver reserve ability. PAB was reported in four out of the 14 studies. The pre-LES versus post-LES comparison included 163 participants. However, there was statistical heterogeneity among these trials (*I*^2^ = 98%). Using the random effects model, results indicate a significant increase in PAB from baseline (MD = 85.84, 95% CI: 41.33–130.34, *P* = 0.0002) (Figure 4).

**Figure 5:**
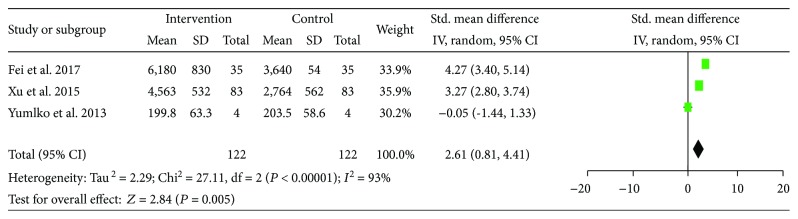
Forest plot for CHE.

CHE was examined using standardized mean differences (SMD) to account for difference in measurement unit among the included studies. The pre-LES versus post-LES comparison included a total of 122 participants. A random effect model was used because the statistical heterogeneity was significant in these studies (*I*^2^ = 93%) and a significant increase in CHE was found after a period of LES (SMD = 2.61, 95% CI: 0.81–4.41, *P* = 0.005) (Figure 5).

**Figure 6:**
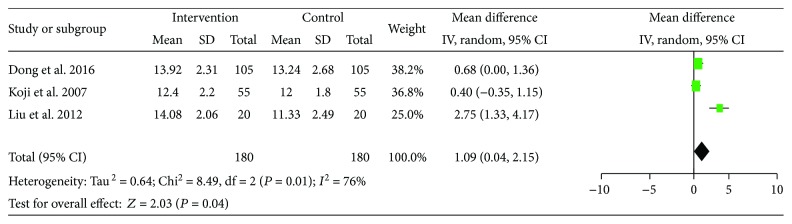
Forest plot for HGB.

HB was examined in three out of the 14 studies. 180 participants were included in the pre-LES versus post-LES analysis. Statistical heterogeneity was observed among these studies (*I*^2^ = 76%), and a random effect model was used for the analysis. The pooled data indicated an increase in HB compared to baseline following LES intervention (MD = 1.09, 95% CI: 0.04–2.15, *P* = 0.04) (Figure 6).

**Figure 7:**
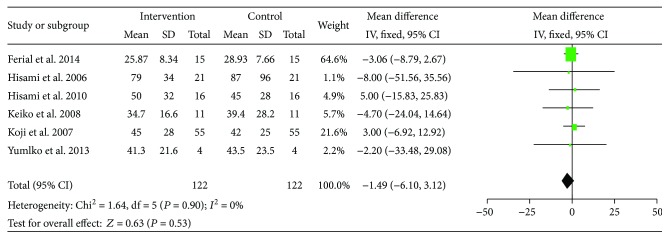
Forest plot for ALT.

**Figure 8:**
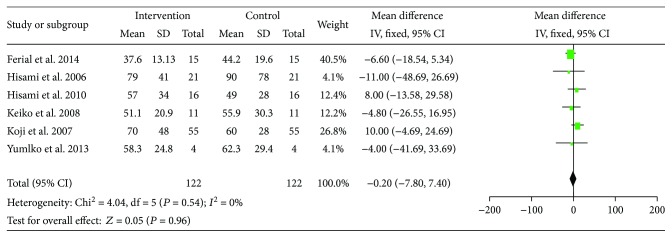
Forest plot for AST.

ALT and AST were reported in six out of the fourteen studies. No substantial heterogeneity was observed among these studies in both ALT (*I*^2^ = 0%) and AST (*I*^2^ = 0%), and a fixed effect model was used for the analysis. The pre-LES versus post-LES analyses included 122 participants, and the pooled MD indicate no decline in ALT and AST after LES administration (MD = −1.49, 95% CI: −6.1–3.12, *P* = 0.53) (MD = −2.0, 95% CI: −7.80 –7.40, *P* = 0.96) (Figures 7 and 8).

**Figure 9:**
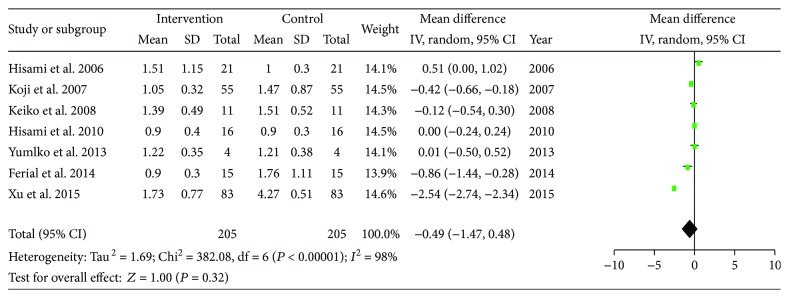
Forest plot for TB.

Bilirubin was examined among seven studies; the pre-LES versus post-LES analyses included 205 participants. There is considerable heterogeneity among these studies (*I*^2^ = 98%). A random effect model was used, and no differences were found compared to baseline (MD = −0.49, 95% CI: −1.47–0.48, *P* = 0.32) (Figure 9).

### 3.3. Energy Metabolism

**Figure 10:**
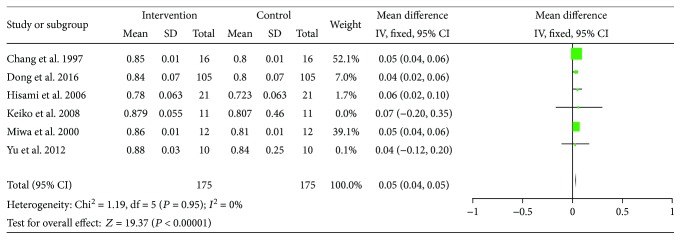
Forest plot for RQ.

Six studies have reported data on RQ. No significant heterogeneity between these studies (*I*^2^ = 0) was found; with the fixed effect model, the pooled MD showed a significant increase in RQ with 1 to 3 weeks of LES administration (MD = 0.05, 95% CI: 0.04–0.05, *P* < 0.00001) (Figure 10).

**Figure 11:**
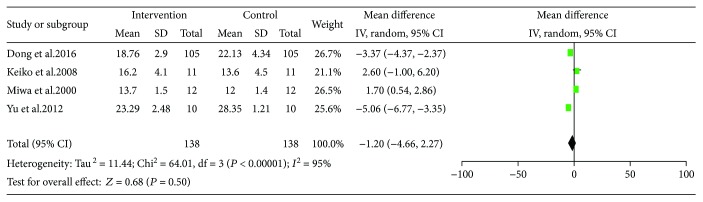
Forest plot for PRO%.

We evaluated the effect of LES on substrate oxidation. Four studies that included 138 participants reported it. A random effect model was used because statistical heterogeneity was significant in protein oxidation rate (*I*^2^ = 84%), carbohydrate oxidation rate (*I*^2^ = 66%), and fat oxidation (*I*^2^ = 95%). The pooled MD for protein oxidation rate showed a trend toward decreasing after LES intake but did not reach statistical significance (MD = −1.20, 95% CI: −4.66–2.27, *P* = 0.50) (Figure 11).

**Figure 12:**
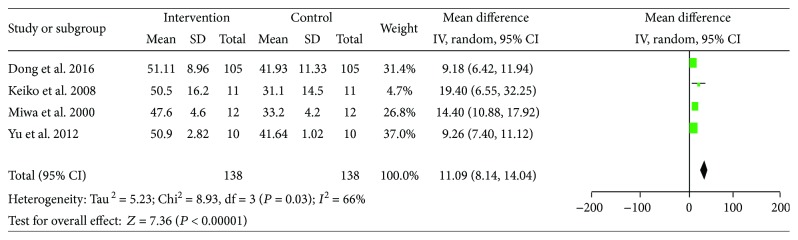
Forest plot for CHO%.

**Figure 13:**
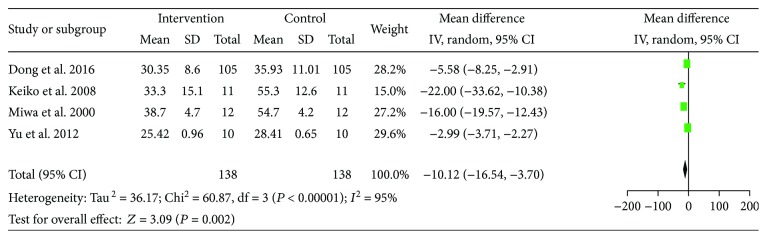
Forest plot for FAT%.

Here, evidence of LES administration improving fuel metabolism was achieved. The pooled MD showed that the utilization of carbohydrate significantly increased (MD = 11.09, 95% CI: 8.14–14.04, *P* < 0.00001) and fat oxidation decrease significantly (MD = −10.12, 95% CI: −16.54 to −3.70, *P* < 0.00001) (Figures 12 and 13). The results implied that the catabolic state of cirrhosis patients improved after LES therapy.

## 4. Discussion

The liver plays a central role in the metabolism of many nutritional elements (carbohydrate, protein, fat, vitamins, and minerals). The metabolism of these nutritional elements is gradually disturbed with progressive chronic liver disease. Characteristic metabolic alterations, including protein energy malnutrition, depleted hepatic glycogen storage and impaired hepatic glycogenolysis, and increased fat oxidation has been found in cirrhotic subjects [25, 26].

The reports on progress made in nutritional science in recent years indicate that LES can lead to a better prognosis and quality of life in cirrhotic patients [27]. However, a quantitative data pooling of clinical evidences on the effect of LES is not obtained.

In this systematic review and meta-analysis, 14 clinical studies, published from 1997 to 2017, with a combined subject population of 478 patients who received LES therapy for at least one week were reviewed and quantitatively analyzed.

This meta-analysis showed that the levels of serum albumin, prealbumin, and cholinesterase were significantly increased with the LES treatment. These biomarkers reflect synthetic metabolism of liver cell. It is reported that CHE activity has an important clinical significance in estimating the prognosis of patients with cirrhosis [28]. Serum albumin provides a better assessment of malnutrition. The levels of ALT, AST, and TB, examined in seven out of the 14 studies, were not significantly different from the baseline when supplying LES in cirrhotic patients. This indicates that bedtime snack may not contribute to liver parenchyma damage of patients with cirrhosis in a short time, even improving the protein synthesis and energy metabolism.

Protein-energy malnutrition is a common characteristic in cirrhotic patients [29]. BCAA supplement served as substrates for protein synthesis, and important regulators of protein synthesis are effective in improving nitrogen balance and finally resulting in better clinical outcomes [30]. Some clinical trials also have demonstrated the effect of BCAAs in patients with hepatic encephalopathy [31].

Our meta-analysis also indicated that both carbohydrate oxidation and fat oxidation were significantly improved. LES reduced the overnight catabolic state in patients with liver cirrhosis. The supplementation of carbohydrate (e.g., rice ball, bread and jam, and oral glucose), BCAA, Chinese herbal, or amazake, given as LES, all improved energy metabolism in liver cirrhosis patients [13, 14, 16, 32]. Nakaya et al. [33] reported that supplement with a BCAA mixture can be used to improve the catabolic state.

Patients with liver cirrhosis usually suffered impaired glucose tolerance. A study in Japan [21] reported that the concomitant use of an *α*-glucosidase inhibitor with LES may improve glucose tolerance and energy metabolism. Aoyama et al. [22] reported that 75 g OGTT (a 75 g oral glucose tolerance test) for the evaluation of glucose tolerance with cirrhosis patients is necessary to determine which patients are best suited for LES administration. Highly individual and specialized management may be required with LES treatment.

LES also improved RQ associated with energy balance, health-related quality of life (HRQOL) scores, and Child–Pugh score. Glass et al. [34] reported that the survival rate was significantly higher in patients with high RQ (>0.85) than in patients with scores below 0.85 with LES treatment. Both Yamanaka-Okumura et al. [12] and Dong et al. [20] have concluded that LES administration was helpful in maintaining higher HRQOL in liver cirrhosis patients. Dong et al. [20] reported that the proportion of Child–Pugh grade A patients increased from 60% to 72.38% and that the proportion of Child–Pugh grade C patients reduced from 8.57% to 1.90% (both *P* < 0.05), with LES nutritional therapy.

Therefore, in 2002, the American Society for Parenteral and Enteral Nutrition (ASPEN) recommended that cirrhotic patients should divide their dietary intake into 4 to 6 meals per day, including LES [35]. The European Society for Clinical Nutrition and Metabolism (ESPEN) advocated a regular daily diet that contained 35–40 kcal/kg/day in energy for cirrhosis patients [36].

There are several limitations in this meta-analysis to be considered. Firstly, most studies included in the meta-analysis were single-center studies; furthermore, the sample size in some of the studies was small. Then, the studies were highly heterogeneous. For the lack of enough detailed data, subgroup analysis stratified by age, sex, and different Child–Pugh classification, which might bring up heterogeneity, could not be carried out. These factors could have introduced an element of bias and affect the results of the meta-analysis. More prospective, multicenter observational studies are required to confirm our findings.

## 5. Conclusion

This meta-analysis indicates that LES could significantly improve malnutrition and correct abnormal fuel metabolism in cirrhotic patients. However, the limited data suggests that it offers no benefit in liver parenchyma damage, without significantly decreasing the level of serum aminotransferase. Based on these results, LES should be considered an appropriate nutrition support for people with cirrhosis.

## Figures and Tables

**Table 1:** Characteristics of the studies included in this meta-analysis.

Study	Year	Country	Design	Sample size	Duration	Age (years)^♦^	Child–Pugh scores	Intervention
Fei et al. [11]	2017	China	RCT	70	4 weeks	54.8 ± 0.69	B and C	150–200 ml herbal cuisine
Yamanaka-Okumura et al. [12]	2010	Japan	RCT	39	12 months	—	A	A high-carbohydrate LES (e.g., a rice ball, a rice cake, and a sweet potato) (200 kcal)
Liu et al. [13]	2012	China	RCT	40	20 days	26–66	C	30 g branched-chain amino acid
Chen et al. [14]	2014	China	RCT	40	6 weeks	49.4 ± 12.7	20 A, 14 B, and 6 C	200 g yogurt and 15 g protein compounds (200 kcal)
Xu et al. [15]	2015	China	RCT	116	4 weeks	—	B and C	200 ml milk
Yamanaka-Okumura et al. [16]	2006	Japan	Case-control study	47	1 week	—	A	Rice ball (200 kcal)
Chang et al. [17]	1997	China	Case-control study	24	—	50 ± 3	A, B, and C	50 g carbohydrate (two slices of bread)
Miwa et al. [18]	2000	Japan	Case-control study	26	1 week	63 ± 2	A, B, and C	250 ml liquid nutrient (250 kcal)
Yu et al. [19]	2012	China	Case-control study	60	2 weeks	42.59 ± 9.67	20 A, 20 B, and 20 C	Carbohydrate (bread)
Dong et al. [20]	2016	China	Pre–post study	105	12 weeks	50.83 ± 8.52	63 A, 33 B, and 9 C	50 g lotus root starch (836.4 KJ)
Keiko et al. [21]	2008	Japan	Pre–post study	11	12 weeks	44–78	3 A, 7 B, and 1 C	Branched-chain amino acid-enriched nutrient mixture
Koji et al. [22]	2007	Japan	Pre–post study	55	3 months	48–85	26 A, 26 B, and 3 C	Branched-chain amino acid-enriched nutrient mixture (210 kcal)
Nagao and Sata [23]	2013	Japan	Pre–post study	4	12 weeks	67.3 ± 5.7	A and B	Amazake (200 kcal)
El-Bassat et al. [24]	2014	Egypt	Case-control study	30	15 days	—	B and C	15 g protein-containing snack (300 kcal)

^♦^Mean age or the range of age.
